# Enhanced histamine-induced itch in diacylglycerol kinase iota knockout mice

**DOI:** 10.1371/journal.pone.0217819

**Published:** 2019-06-05

**Authors:** Victoria Brings Bartsch, Jesse K. Niehaus, Bonnie Taylor-Blake, Mark J. Zylka

**Affiliations:** 1 Department of Cell Biology and Physiology, The University of North Carolina at Chapel Hill, Chapel Hill, North Carolina, United States of America; 2 UNC Neuroscience Center, The University of North Carolina at Chapel Hill, Chapel Hill, North Carolina, United States of America; University College London, UNITED KINGDOM

## Abstract

Subsets of small-diameter dorsal root ganglia (DRG) neurons detect pruritogenic (itch-causing) and algogenic (pain-causing) stimuli and can be activated or sensitized by chemical mediators. Many of these chemical mediators activate receptors that are coupled to lipid hydrolysis and diacylglycerol (DAG) production. Diacylglycerol kinase iota (DGKI) can phosphorylate DAG and is expressed at high levels in small-diameter mouse DRG neurons. Given the importance of these neurons in sensing pruritogenic and algogenic chemicals, we sought to determine if loss of DGKI impaired responses to itch- or pain-producing stimuli. Using male and female *Dgki*-knockout mice, we found that *in vivo* sensitivity to histamine—but not other pruritogens—was enhanced. In contrast, baseline pain sensitivity and pain sensitization following inflammatory or neuropathic injury were equivalent between wild type and *Dgki*^-/-^ mice. *In vitro* calcium responses in DRG neurons to histamine was enhanced, while responses to algogenic ligands were unaffected by *Dgki* deletion. These data suggest *Dgki* regulates sensory neuron and behavioral responses to histamine, without affecting responses to other pruritogenic or algogenic agents.

## Introduction

Chronic itch (pruritis) and chronic pain can drastically impact daily function, often hindering work performance and severely impairing quality of life [1, 2]. Current treatments do not adequately alleviate symptoms [3, 4] and come with serious side effects [5]. Itch- and pain-sensing neurons, whose cell bodies lie in the dorsal root ganglia (DRG), respond to pruritogenic and noxious stimuli in the periphery and transduce these signals to the central nervous system for sensory processing [4]. Pruritogenic compounds released by immune cells activate itch fibers that innervate the skin [6]. While this activation enables recognition of allergens, many pruritis patients suffer from aberrant, unprovoked activity of itch fibers, leading to extreme scratching behavior. Similarly, algogenic compounds released following injury to nerves or tissues throughout the body can activate and sensitize peripheral pain fibers, shifting patients toward a state of pain hypersensitivity that can persist after the injury has healed [7]. Somatosensory neurons are the primary responders to pruritogenic and noxious stimuli, and their dysfunction can cause chronic itch or chronic pain. Characterizing the function of signaling mediators expressed in DRG neurons may reveal candidate molecules to target for the treatment of itch and/or pain.

Although itch and pain are distinct sensations, the neuronal populations that respond to these stimuli are largely overlapping [4]. Activation of receptor tyrosine kinases and Gα_q_-protein-coupled receptors on itch- and pain-sensing neurons induces the production of diacylglycerol (DAG), an important lipid signaling molecule in the DRG [8]. Diacylglycerol kinases (DGKs) can terminate DAG signaling by phosphorylating DAG to produce phosphatidic acid (PA). DAG and PA modulate neuronal function by altering the activity of numerous downstream effectors [9–11]. Lipid kinases sit at a convergence point in signaling pathways that regulate pain signaling [12]. DAG and PA are produced downstream of multiple receptors, and both of these second messengers have important signaling functions in sensory neurons. Therefore, defining how DGKs regulate the balance between these two lipids could have significant implications for our understanding of the molecular mechanisms of itch and pain. Indeed, disrupting activity of a lipase that converts DAG to monoacylglycerol attenuates pain responses [13]. Thus, altering the metabolism of lipids in this network can affect somatosensation.

Here, we focus on DGK iota (*Dgki*), which is expressed in peripheral somatosensory neurons. The role of *Dgki* in somatosensation has never been studied. Deletion of *Dgki* in mice was previously found to reduce Ras activity in primary embryonic fibroblasts and diminish Ras-dependent skin tumor formation [14]. Others found that *Dgki* positively regulates metabotropic glutamate receptor-dependent long-term depression at Schaffer collateral-CA1 synapses in neonates, but not in adults [15]. Litters raised by *Dgki*^-/-^ dams had a slightly reduced survival rate [16]. *Dgki*-knockout mice showed a latency to habituate to an open field in one study [15], but an absence of an open field phenotype was also reported [16]. No other phenotypes related to locomotion, depression, anxiety, or learning were found.

Here, we characterized the role of *Dgki* in pruritogenic and noxious sensory behaviors in male and female mice. Additionally, we examined the responses of cultured DRG neurons from these animals to pruritogenic and algogenic stimuli.

## Materials and methods

### Mice

All procedures used in this study were approved by the Institutional Animal Care and Use Committee at the University of North Carolina at Chapel Hill. We acquired a *Dgki*^-/-^ mouse line [14], which was on a mixed 129 and C57BL/6 background. All data presented here were from mice that were backcrossed with C57BL/6J mice for at least five generations. We performed PCR with genomic DNA isolated from tail clips to confirm genotypes of our mice, as described previously [14].

Mice were maintained on a 12 h:12 h light:dark cycle and given food (Teklad 2020X, Envigo, Huntingdon, United Kingdom) and water *ad libitum*. Mice were group housed with 3 to 5 mice per cage. Cages had Bed-o’Cobs bedding (Andersons Lab Bedding, Maumee, Ohio). Given a prior study showing how bedding can influence mechanical sensitivity post-injury [17], for the spared nerve injury (SNI) experiments, the bedding was 1/8-inch diameter (8B, Andersons Lab Bedding). During all other experiments, the bedding was 1/4-inch diameter (4B, Andersons Lab Bedding).

### Immunohistochemistry

Three adult (Postnatal day 90) male wild-type (WT) mice and one adult *Dgki*^-/-^ mouse were deeply anesthetized with pentobarbital (50 mg/kg) and perfused with ice-cold PBS followed by ice-cold 4% paraformaldehyde (PFA) in 0.1 M phosphate buffer (13.8 g/L Na_2_HPO_4_-H_2_O, 26.8 g/L Na_2_HPO_4_-7H_2_O, pH 7.4). DRGs were removed and placed into the same fixative for 4 h at 4°C. DRGs were moved to 30% sucrose in 0.1 M phosphate buffer at 4°C for cryoprotection and later embedded in TFM (Tissue Freezing Medium; TFM-5, General Data, Cincinnati, OH). Sections of 25-μm thickness were collected onto SuperFrost Plus glass slides (12-550-15, Thermo Fisher Scientific, Waltham, Massachusetts) and stored at -20°C until staining. Sections were rehydrated with PBS, washed with a solution of Tris-buffered saline with Triton X-100 (TBST; 0.05 M Tris, 2.7% w/v NaCl, 0.3% v/v Triton-X 100, pH 7.6), and blocked in 10% normal donkey serum (S30, EMD-Millipore, Burlington, Massachusetts) in TBST (NDS/TBST) for 1 h at room temperature (approximately 23°C). Sections were then incubated in primary antibody cocktails in NDS/TBST overnight at room temperature. Primary antibodies used were Mouse anti-DGKI (1:300; MAB6435, R&D Systems, Minneapolis, Minnesota, RRID: AB_10730703), Guinea-pig anti-NeuN (1:400; ABN90P, EMD-Millipore; RRID: AB_2341095), Rabbit anti-NF200 (1:900; N4142, Sigma-Aldrich, St. Louis, Missouri, RRID: AB_477272), Rabbit anti-TH (1:100; AB152, EMD-Millipore, RRID: AB_390204), Rabbit anti-CGRP (1:100; BML-CA1134, Enzo Life Sciences, Farmingdale, New York, RRID: AB_2068527), and Rabbit anti-TRPV1 (1:300; ACC-030, Alomone Labs, Jerusalem, Israel, RRID: AB_2313819). The following day, sections were rinsed in TBST and blocked with NDS/TBST for 30 min before incubation in a secondary antibody cocktails in NDS/TBST for 6 h at room temperature. All secondary antibodies were used at 1:500 and included Goat anti-mouse IgG_2a_-Alexa 488 (A21131, Thermo Fisher Scientific, RRID: AB_2535771), Donkey anti-rabbit IgG-Alexa 568 (A10042, Thermo Fisher Scientific, RRID: AB_2534017), and Donkey anti-guinea-pig IgG-Alexa 647 (706-605-148, Jackson ImmunoResearch Laboratories, West Grove, Pennsylvania, RRID: AB_2340476). In some experiments, Isolectin GS-IB4-Alexa 647 (1:200; I32450, Thermo Fisher Scientific) was added to the secondary antibody cocktail. Sections were rinsed with TBST and PBS and the slides were coverslipped with FluoroGel mounting medium (17985–10, Electron Microscopy Sciences, Hatfield Pennsylvania). Imaging was performed on a Zeiss LSM 710 confocal laser scanning microscope. For quantification of DGKI+ neuron size and colocalization of DGKI with TH, NF200, TRPV1, CGRP, or IB4, sections from all three WT animals were analyzed. Images were processed using Fiji software for input into CellProfiler analysis software, which identified and counted cells expressing DGKI and/or TH, NF200, TRPV1, CGRP, and IB4 and calculated the average radius of each DGKI+ cell. For DGKI/TH colocalization, 30 sections were stained and analyzed; for DGKI/NF200, 24 sections; for DGKI/TRPV1, 21 sections; for DGKI/CGRP, 24 sections; and for DGKI/IB4, 24 sections. Each section had at least 18 DGKI-positive (DGKI+) cells. A total of 5,870 DGKI+ cells, from all costaining conditions, were measured for average radius, from which soma diameter was calculated.

### Western blotting

Frontal cortex, spinal cord, and DRG tissue was dissected (in that order) from 4-week-old wild-type (WT) and *Dgki*^-/-^ male and female mice, placed in ice-cold lysis buffer (50 mM Tris pH 7.4, 150 mM NaCl, 1 mM EDTA pH 8.0, 1% v/v Triton-X100, 1 mM phenylmethanesulfonyl fluoride, 1 mM sodium deoxycholate, 1× cOmplete Mini EDTA-free Protease Inhibitor Cocktail [4693159001, Roche, Basel, Switzerland]) for 10 min. Following sonication on ice for 10 s, lysates were centrifuged at 10,000 × g at 4°C for 15 minutes to separate the debris. Protein (30 μg) from each lysate was separated on a 4–20% SDS/PAGE gel (456–1094, Bio-Rad, Hercules, California) and transferred to a 0.2 μm nitrocellulose membrane (1620095, Bio-Rad). Following 1 h of blocking in a solution of 5% w/v milk (170–6404, Bio-Rad) in Tris-buffered saline with Tween 20 (TBST; 100 mM Tris pH 7.5, 165 mM NaCl, 0.1% v/v Tween 20) at room temperature, membranes were incubated overnight at 4°C with primary antibodies for β-actin (1:3,000 mouse anti- β-actin monoclonal antibody; ab6276, Abcam, Cambridge, United Kingdom, RRID: AB_2223210) and DGKI (1:1,000 rabbit anti-DGKI polyclonal antibody; LS-C118721, Lifespan Biosciences, RRID: AB_10798112) in a solution of 5% w/v bovine serum albumin (BSA; A3912, Sigma-Aldrich) in TBST. Blots were probed with secondary antibodies of 1:10,000 IRDye 680RD-conjugated goat anti-mouse polyclonal antibody (925–68070, LI-COR Biosciences, Lincoln, Nebraska, RRID: AB_2651128) and 1:10,000 IRDye 800RD-conjugated donkey anti-rabbit antibody (926–32213, LI-COR, RRID: AB_621848) in 5% w/v milk in TBST for 2 h at room temperature. Blots were washed with TBST between each step. Blots were imaged on a LI-COR Odyssey system.

### Behavior

All behavior assays were performed on 2- to 4-month-old mice during the light phase of the light:dark cycle. For each assay, mice within each cohort were tested in a random order that varied between days. After testing each mouse for itch or baseline pain responses, mice were kept separate from the mice that had not yet been tested in that assay, to eliminate the potential for pain empathy responses [18]. For all behavior assays, the experimenters were blinded to the genotype.

Sensory behavior assays were performed in a mesh platform apparatus or a glass platform apparatus, except for the tail immersion and clothespin assays. The mesh platform apparatus consisted of a 28 × 46 cm sheet of stainless-steel mesh elevated 28 cm from the bench surface. Mice were placed on top of the mesh platform, and each mouse was enclosed in an individual 9 × 9 × 11 cm 5-sided transparent plastic box. For the glass platform apparatus, we used an 86 × 35 cm pane of 0.6 cm thick glass elevated 21 cm from the bench. Mice were placed in square acrylic enclosures of 13 cm height that were separated with dividers into 4 equally-sized 10 × 10 cm chambers. Lids of the acrylic chambers had 3-cm-diameter holes, covered with copper wire mesh to prevent escape.

Itch, cold plantar, and Hargreaves assays were tested in the glass platform apparatus. Cotton swab and filamentous von Frey assays were tested in the mesh platform apparatus. Mice were acclimated to these apparatuses for 2 hours per day for at least 5 days prior to testing. On the day of testing itch responses, mice were acclimated to test chambers for 30 minutes prior to injection, and only one animal was tested at a time. On the day of testing cotton swab, cold plantar, Hargreaves, and von Frey, mice were acclimated to the test chamber for 1 h prior to testing, and all mice in the cohort were tested together.

#### Itch assay

We used an acute scratching assay to test itch responses in WT and *Dgki*^-/-^ males and females. We tested histamine, chloroquine, and β-alanine. Histamine is released by mast cells after allergen exposure and is a target of many antipruritic drugs [6, 19]. Chloroquine is an anti-malaria drug that causes severe pruritis in black Africans [20]. β-alanine is a supplement commonly taken by body builders to regulate muscular pH levels that causes a minor itch side effect [21].

Following a 30-minute acclimation period, mice were injected with 50 μL of pruritogen that was dissolved in 0.9% w/v NaCl, made fresh on the day of testing, and kept on ice throughout the day. The 50-μL injection contained 500 μg β-alanine (A9920, Sigma-Aldrich), 500 μg histamine (H7250, Sigma-Aldrich), or 200 μg chloroquine (C6628, Sigma-Aldrich). After injecting a pruritogen subcutaneously at the nape of the neck using a 13-mm 30-gauge needle (305106, BD, Franklin Lakes, New Jersey) attached to a 50-μL glass syringe (Model 705, Hamilton, Reno, Nevada), we counted scratching bouts at the site of injection performed by each mouse in 5-minute intervals over 30 minutes.

WT and *Dgki*^-/-^ males and females were tested together, in two cohorts. The first cohort had 10 mice each of WT males, *Dgki*^-/-^ males, WT females, and *Dgki*^-/-^ females; the second cohort had 12 WT males, 11 *Dgki*^-/-^ males, 10 WT females, and 8 *Dgki*^-/-^ females. All three pruritogens were tested in each mouse. The first cohort was tested in the order of increasing pruritogen strength (β-alanine, histamine, chloroquine) to decrease the likelihood that their skin would be irritated by scratching induced by the previous pruritogen. Upon finding an increased response to histamine in *Dgki*^-/-^ mice in the first cohort, the second cohort was tested in the following order: β-alanine, chloroquine, histamine. The same effects were found in each cohort individually, so we combined the data from both cohorts here. Mice were given at least 48 hours to recover from injections of β-alanine, and at least 72 hours to recover from injections of histamine or chloroquine.

#### Acute pain sensitivity assays

Mice were tested on each baseline pain assay at least twice, on separate days, and the responses for an individual animal were averaged. Mice were given at least one hour to recover after each test. In a single day, mice were tested on 2 to 5 baseline pain assays. The assays were run in a random order that varied between days. The same two cohorts of animals used for the itch assays (see Itch assay methods for genotype/sex composition of each cohort) were tested in the tail immersion, clothespin, cotton swab, cold plantar, and hot plate assays. The first cohort of 40 mice was tested on -10°C, 46.5°C, and 49°C tail immersion and clothespin assays. The second cohort of 41 mice was tested on -10°C, 46.5°C, and 49°C tail immersion; clothespin; cotton swab; and hot plate assays. Three *Dgki*^-/-^ males had damaged tails, so they were excluded from tail assays (tail immersion and clothespin). One *Dgki*^-/-^ male and one *Dgki*^-/-^ female squirmed during the -10°C tail immersion assay, giving ambiguous responses that were excluded from the data.

**Tail immersion**—To measure thermal sensitivity [22, 23], a 75% ethanol solution was cooled to -10°C or a water bath was heated to 46.5°C or 49°C. Each mouse was gently wrapped in a towel and inverted, submerging approximately half of the tail, and the latency for the mouse to flick their tail was measured. Without a response, tails were removed from -10°C after 60 s, 46.5°C after 40 s, and 49°C after 20 s.

**Clothespin**—To measure mechanical sensitivity [24], we measured the latency for a mouse to attempt to remove a 2.5-cm long miniature clothespin (3438, Bazic Products, Los Angeles, California) attached to the tail, approximately 1 cm from the distal tip. Assays were performed in an empty 17 × 28 × 12.5 cm plastic cage (Allentown Caging, Allentown, New Jersey) with no lid. The time for a mouse to bite or attempt to remove the clothespin was measured. The clothespin was removed as soon as the mouse responded, or after 20 s if there was no response.

**Cotton swab**—To determine if *Dgki* loss affects large-diameter, low-threshold mechanosensory neurons of the DRG [25], we assessed responses to a light touch via a “fluffed out” cotton swab brushed across the hindpaw. The cotton swab was brushed from heel to toe, and any movement of the paw was counted as a response. Each paw was tested 5 times (total of 10 tests per mouse). After testing each paw once for each animal, the mice were given 10 min to settle before testing again. We alternated starting testing on either the left or right hindpaw.

**Cold plantar**—To examine sensitivity to cold, we measured withdrawal latency to applications of dry ice to the hindpaw [26]. Dry ice was packed into a 3-mL syringe (209657, BD) modified to expose a 0.8-cm-diameter cross section and pressed against the glass directly under the hindpaw. Without a response, the stimulus was removed after 20 s. Each hindpaw was tested two to three times, with at least 30 min rest between tests on a single paw. We alternated starting testing on either the left or right hindpaw.

**Hot plate**—To examine sensitivity to heat, we measured latency to respond to placement of the mouse onto a 55°C hot plate [27], using a Hot Plate Analgesia Meter (Series 8 Model 29, IITC Life Science Inc., Woodland Hills, California). Mice were placed on the surface of the hot plate, within an acrylic cylinder with an inner diameter of 10 cm and a height of 15.4 cm. The latency to demonstrate a painful response (licking or rapidly flicking either hindpaw or jumping) was timed. The mouse was removed from the plate after responding, or after 30 s if there was no response.

**Hargreaves**—To examine sensitivity to heat, we measured withdrawal latency to applications of heat via a focused beam of light to the hindpaw [28], using an infrared radiometer (72–6703, Harvard Apparatus, Holliston, Massachusetts) light source set to an intensity of 30 mW/cm^2^. Without a response, the stimulus was removed after 20 s. Each hindpaw was tested two to three times, with at least 30 min rest between tests on a single paw. We alternated starting testing on either the left or right hindpaw.

**Filamentous von Frey**—To examine mechanical pain, we used von Frey filaments. Pressure is applied to the plantar surface of the hindpaw using a long, thin filament until either the filament bends or the mouse withdraws its paw [29]. Eight filaments with bend thresholds of 0.407, 0.692, 1.2, 1.5, 2.04, 3.63, 5.5, and 8.5 g (Research Designs Inc.) were tested, in that order. Each filament was tested five times on each hindpaw (alternating left and right for each test). The percentage of total hindpaw withdrawals (out of five) was calculated to assess sensitivity to mechanical stimuli. Mice were allowed to rest for at least 2 minutes between tests of an individual filament and a minimum of 15 minutes between filaments to ensure withdrawals were due to mechanical nociception rather than overstimulation.

#### Chronic pain models

**Inflammatory injury**—We modeled inflammatory sensitization by injecting 30 μL of complete Freund’s adjuvant (CFA; 855828, MP Biomedicals, Santa Ana, California) into the plantar surface of the left hindpaw, using a 13-mm 27-gauge needle (305109, BD) attached to a 50 μL glass syringe (Model 705, Hamilton) [30]. Responses of both the ipsilateral and contralateral hindpaws to heat in the Hargreaves test were measured at baseline (0 to 3 days prior to CFA injection) and on Days 1, 2, 3, and 7 after CFA injection. Males were additionally tested on Day 4 and females on Day 5. Observation days were chosen to observe development of and recovery from CFA-induced inflammatory sensitization. Forty mice were assayed in the CFA injury model: 10 mice each of WT males, *Dgki*^-/-^ males, WT females, and *Dgki*^-/-^ females.

**Neuropathic injury**—Neuropathic sensitization was induced using the spared nerve injury (SNI) model of neuropathic pain, in which two of the three major branches of the sciatic nerve are ligated and transected while the third is left intact [31, 32]. Animals were anesthetized via isoflurane insufflation. Once unconscious (confirmed by toe pinch reflex), hair was removed from the left hindleg before being stabilized via surgical pins (tissue was not pierced). The incision site was cleaned with three alternating washes of chlorohexidine and iodine solution. A 2-cm incision was made through the skin at the mid-thigh level. The biceps and semitendinosus muscles were gently teased apart using forceps, exposing the sciatic nerve. The peroneal and sural nerves were tightly ligated with 6–0 silk suture while the tibial nerve was left intact. The ligated nerves were then transected distal to the ligature and 2 mm of each branch distal to the nerve stump were removed to confirm complete transection. The muscles were repositioned back over the nerve and the skin wound closed via surgical clips. The animals were then placed in a cage on a heating pad until they woke before being returned to their home cage. Total time unconscious was approximately 20 minutes. Animals were monitored daily to ensure the wound remained sealed and clean. Surgical clips were removed 6 days post-surgery. Responses of both the ipsilateral and contralateral hindpaws to mechanical stimulation in the filamentous von Frey assay were tested at baseline (1 to 3 days prior to SNI surgery), and Days 1, 7, and 14 after surgery. Due to the length of time of each surgery, the 40 total mice were tested in 4 cohorts. The first cohort had 7 WT and 6 *Dgki*^-/-^ males, the second had 5 WT and 5 *Dgki*^-/-^ males, the third had 5 WT and 3 *Dgki*^-/-^ females, and the fourth had 5 WT and 4 *Dgki*^-/-^ females.

### Primary DRG neuron cultures

Similar to previous work [33], DRGs were dissected from 4- to 8-week-old male and female WT and *Dgki*^-/-^ mice and placed into a 1.5-mL microcentrifuge tube containing 1 mL of calcium- and magnesium-free Hank’s balanced salt solution (HBSS; 14175–145, Gibco, Grand Island, New York; 400 mg/L KCl, 60 mg/L KH_2_PO_4_, 350 mg/L NaHCO_3_, 8 g/L NaCl, 48 mg/L Na_2_HPO_4_, 1 g/L D-glucose) on ice. After a quick spin, HBSS was gently removed and replaced with 1 mL of a solution of 2 mg/mL collagenase (CLS1, Worthington Biochemical, Lakewood, New Jersey) and 5 mg/mL dispase (17105–041, Gibco) in HBSS. The tube was placed in a 37°C water bath for 30 minutes, inverting every 5 minutes. After 20 minutes, DRGs were triturated with a flame-polished glass Pasteur pipette, then returned to the water bath. After 30 minutes, the 1-mL DRG suspension was mixed with 1 mL of 37°C supplemented media containing Neurobasal A (10888, Gibco), 0.5% B-27 (17504, Gibco), 100 U/mL penicillin/streptomycin (15140, Gibco), 2 mM L-glutamine (25030, Gibco), 50 ng/mL glial derived neurotrophic factor (GF030, EMD-Millipore), and 25 ng/mL nerve growth factor (01–125, EMD-Millipore). The 2-mL DRG suspension was triturated with a fresh Pasteur pipette and placed on a pre-moistened 70 μm filter (22-363-548, Thermo Fisher). The filtrate was spun at 1000 × g for 5 minutes at room temperature. The supernatant was carefully removed, and the pellet was resuspended in 37°C supplemented media with a fresh Pasteur pipette. Using a pipette with a plastic tip, 90 μL of the cell suspension was plated onto each coverslip (354087, Corning, Corning, New York), prewarmed to 37°C. Coverslips were 12 mm in diameter, precoated with poly-D-lysine and laminin, and each placed in a well of a 24-well plate. After 1–2 h, 410 μL of 37°C supplemented media was added to each well. Cells were kept in a 37°C incubator with 5% CO_2_ and used for calcium imaging within 24 hours. For each preparation, DRGs from one WT and one *Dgki*^-/-^ mouse (age- and sex-matched) were dissected and cultured concurrently and were plated at equal densities on 8–12 coverslips/mouse.

### Calcium imaging

Similar to previous work [10, 33], on the day of imaging cells were washed 2 times with assay buffer: Hanks’ balanced salt solution (14025–126, Thermo Fisher; 140 mg/L CaCl_2_, 100 mg/L MgCl_2_-6H_2_O, 100 mg/L MgSO_4_-7H_2_O, 400 mg/L KCl, 60 mg/L KH_2_PO_4_, 350 mg/L NaHCO_3_, 8 g/L NaCl, 48 mg/L Na_2_HPO_4_, 1 g/L D-glucose) supplemented with 2.4 g/L HEPES, 2 g/L D-glucose, and 0.1% w/v fatty acid–free BSA (A6003, Sigma-Aldrich), at pH 7.3. Cell were then incubated with 2 μM Fura-2, AM (F1221, Invitrogen) in 0.02% Pluronic F-127 (P-3000MP, Invitrogen) in assay buffer for 1 h at room temperature. Cells were washed 3 times with assay buffer, then maintained at room temperature for 30 min before imaging. Assays were performed on an Eclipse Ti microscope (Nikon, Tokyo, Japan) with a CFI Plan Fluor 20x objective (Nikon) and a DG-4 light source (Sutter, Novato, California). Cells were alternately excited for 500 ms at 340 nm and for 250 ms at 380 nm. Emission was measured at 510 nm and recorded using a Clara DR-328G-C01-SIL CCD camera (Andor, Belfast, United Kingdom) and NIS Elements imaging software (Nikon). During imaging, coverslips were placed in a chamber (Model RC-26GLP, Warner Instruments, Hamden, Connecticut) mounted on microscope stage adapter and were perfused with 30°C buffers (heated with SH-27B inline heater, Warner Instruments) at a flow rate of 4.2 mL/min (using a Minipuls 3 Peristaltic Pump, Gilson, Middleton, Wisconsin). After collecting 90 s of baseline Fura-2 ratios while perfusing with assay buffer, cells were perfused with agonist for 90 s, followed by assay buffer for 180 s, then 100 mM KCl for 30 s to test for neuron health. Agonists and 100 mM KCl solutions were made with assay buffer.

The agonists used were 100 μM histamine, 1 μM capsaicin, 10 μM lysophosphatidic acid (LPA), or 100 μM uridine triphosphate (UTP). Histamine activates the H1 receptor on sensory neurons to induce itch responses [19]. Capsaicin is the chemical ligand for the ion channel TRPV1 (transient receptor potential vanilloid 1), an important receptor for itch and pain signaling [19, 34]. Lysophosphatidic acid acts on LPA_1_, LPA_2_, and LPA_3_ receptors (Gα_q_-GPCRs) and induces nociception in peripheral neurons [35, 36]. Uridine triphosphate binds to P2Y receptors and activates and sensitizes small-diameter DRG neurons [37, 38]. We used histamine because of the *in vivo* scratching phenotypes we observed in response to histamine injection. We used capsaicin, LPA, and UTP for algogens because their receptors are expressed in a relatively high percentage of rodent DRG neurons [37, 39, 40].

For analysis, we calculated the Fura-2 ratio (ratio of the emission following excitation at 340 nm/380 nm). We excluded cells that had high baseline Fura-2 ratios (>1.0) or failed to respond to KCl (did not reach ratio of at least 1.0 during KCl exposure). To normalize to baseline, the 60 s of Fura-2 ratios measured immediately preceding agonist exposure were averaged for a given cell, and that average was subtracted from each data point for that cell throughout the whole assay period. Only cells that responded to agonist (had a minimum increase in Fura-2 ratio of 0.1 over baseline average for 2 consecutive time points during period of agonist exposure) were included in our analyses. Area-under-the-curve (AUC) values were calculated on a cell-by-cell basis, using the baseline-normalized values, for the 90-s period of agonist exposure.

DRG cultures from one WT and one *Dgki*^-/-^ mouse (cultured concurrently) were tested each day, alternating coverslips in a random order. For histamine, we used 4 male and 3 female mice each for WT and *Dgki*^-/-^ (14 mice total); here, we present responses from the 114 WT neurons and 124 *Dgki*^-/-^ neurons that were activated by histamine. For capsaicin, we used 2 males of each genotype, and we present the 110 WT and 86 *Dgki*^-/-^ neurons that responded to capsaicin. For LPA, we used 1 male and 1 female of each genotype, and we present the 116 WT neurons and 93 *Dgki*^-/-^ neurons that responded to LPA. For UTP, we used 4 males of each genotype, and we present the 116 WT neurons and 165 *Dgki*^-/-^ neurons that responded to UTP.

### Analysis of microarray data

The *Dgki* expression profile data generated via microarray previously was accessed through BioGPS (dataset “GeneAtlas MOE430, gcrma” and probe set “1439986_at”) [41–43]. For the present study, expression level of *Dgki* for each tissue was normalized to the median expression level for all tissues, and averages were calculated of duplicate samples. The expression levels were ranked highest to lowest, and the 10 tissues with the highest expression are shown here.

### Statistics

Data were analyzed with Prism version 7.04 (GraphPad Software Inc., La Jolla, California). WT vs. *Dgki*^-/-^ responses (by sex) in all baseline pain assays were tested for significance using two-tailed t-tests with Welch’s correction, as were WT vs. *Dgki*^-/-^ AUC responses in calcium activity assays. For each sex, scratching responses to pruritogens, Hargreaves responses in the CFA model, and von Frey responses in the SNI model were compared between WT and *Dgki*^-/-^ using Sidak's multiple comparisons tests used for pairwise comparisons within each 5-min time bin (pruritogens), day (CFA), or filament (SNI) within two-way repeated measures analysis of variance (ANOVA) tests. The proportion of DRG neurons responding to each agonist in the calcium imaging assays was compared between genotypes with a binomial test.

## Results

### *Dgki* is highly expressed in pruriceptive and nociceptive DRG neurons

**Fig 1:**
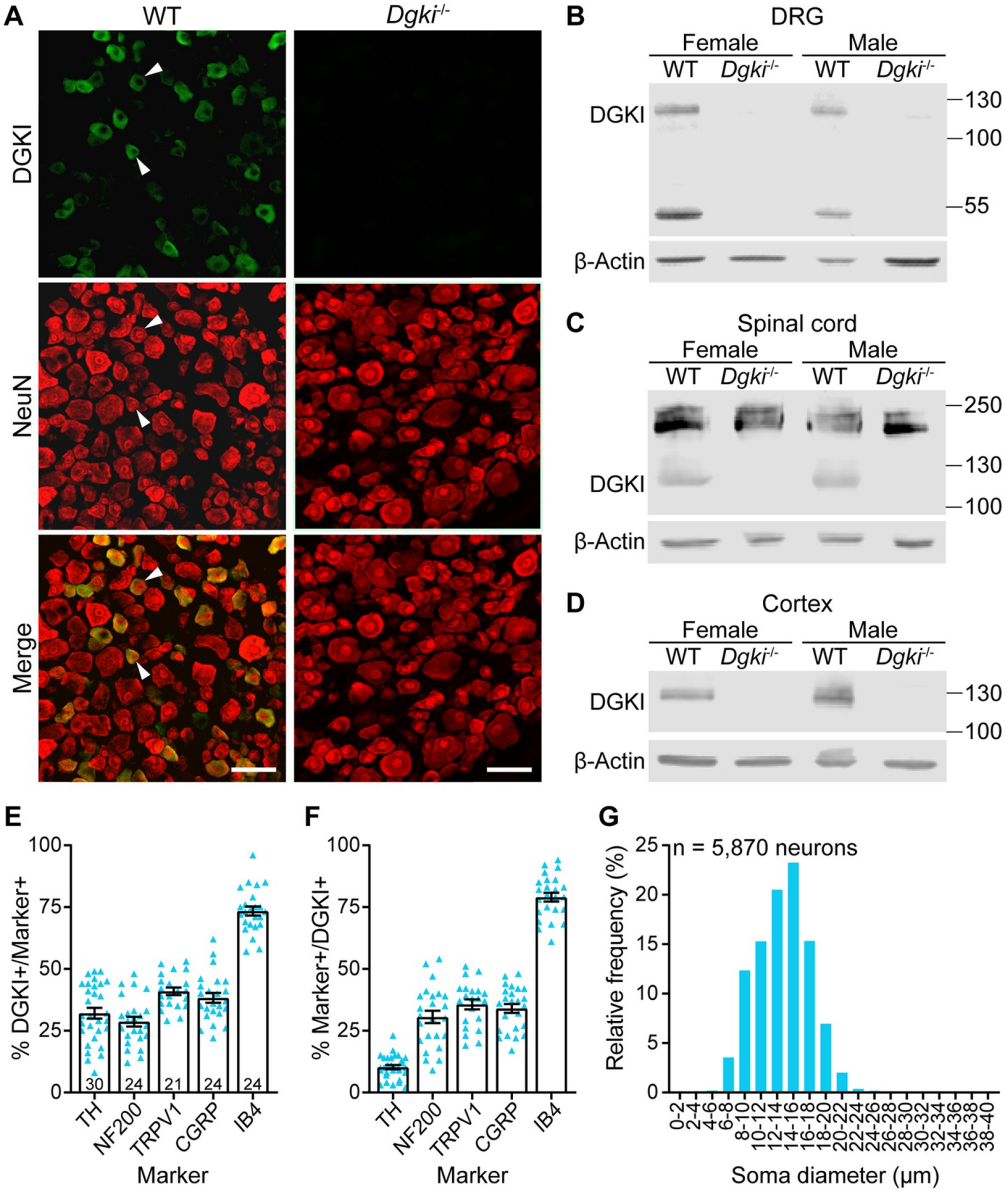
DGKI is enriched in peripheral nociceptors and is absent in neuronal tissues of *Dgki*-knockout mice. (A) Protein immunohistochemical staining shows that DGKI protein is cytoplasmic in small-diameter (arrowheads) WT mouse DRG neurons and is absent in *Dgki*^-/-^ mouse DRG neurons. Scale bars = 50 μm. (B-D) Immunoblotting with lysates from female and male WT and *Dgki*^-/-^ mouse DRG (B), spinal cord (C), and cortex (D) showing that genetic deletion of *Dgki* eliminates the DGKI protein. DGKI and β-actin bands paired together in each panel are from the same blot. (E-F) Analysis of immunohistochemical staining in WT mouse DRG neurons reveals the percent of neurons expressing different markers that also express DGKI (E) and the percent of DGKI-expressing neurons that also express different markers (F). (G) Histogram showing the soma diameter distribution of DRG neurons expressing DGKI. Number of sections plotted in (E) and (F) labeled on graph in (E). Graphs in (E) and (F) show mean ± SEM.

Whereas others have examined the role of *Dgki* in the brain, no one has yet looked at the function of *Dgki* in the DRG, which is the neuronal tissue in which *Dgki* is most highly expressed in mice (Table 1) [41]. Based on single-cell RNA-sequencing of mouse DRG, *Dgki* is expressed in DRG neurons that express genes important for pruriception and nociception, but is expressed at low to undetectable levels in all other subclasses [44]. DRG neuron subclasses can generally be distinguished based on size; itch- and pain-sensing neuronal cell bodies typically have small diameters [45]. Immunostaining of mouse DRG (Fig 1A) revealed that DGKI is expressed in small-diameter neurons (Fig 1G), consistent with its colocalization with IB4 (Fig 1E and 1F), a marker of small-diameter neurons. Additionally, DGKI localizes to the cytoplasm (Fig 1A), consistent with a prior study [46].

**Table 1:** *Dgki* is highly expressed in neuronal tissues and mast cells.

Tissue	Relative *Dgki* expression
Mast cells	27.02
Dorsal root ganglia	12.59
Dorsal striatum	8.74
Olfactory bulb	3.75
Nucleus accumbens	3.52
Hippocampus	3.44
Spinal cord	3.10
Cerebral cortex	3.05
Natural killer cells	2.82
Amygdala	2.22

BioGPS microarray data reveal the murine tissues with the highest expression levels of *Dgki*.

### Disrupting *Dgki* eliminates DGKI expression in mouse DRG

With the availability of a previously-generated *Dgki*-knockout mouse [14], we were able to investigate how *Dgki* loss affects somatosensensory behaviors. We confirmed absence of DGKI in the DRG via immunohistochemical staining (Fig 1A) and showed loss of the expected band at approximately 120 kDa representing DGKI protein in the DRG of male and female *Dgki*^-/-^ mice (Fig 1B). We confirmed loss of DGKI in *Dgki*^-/-^ spinal cord and cerebral cortex, as well (Fig 1C and 1D).

### *Dgki* loss in male and female mice enhances behavioral response to histamine

**Fig 2:**
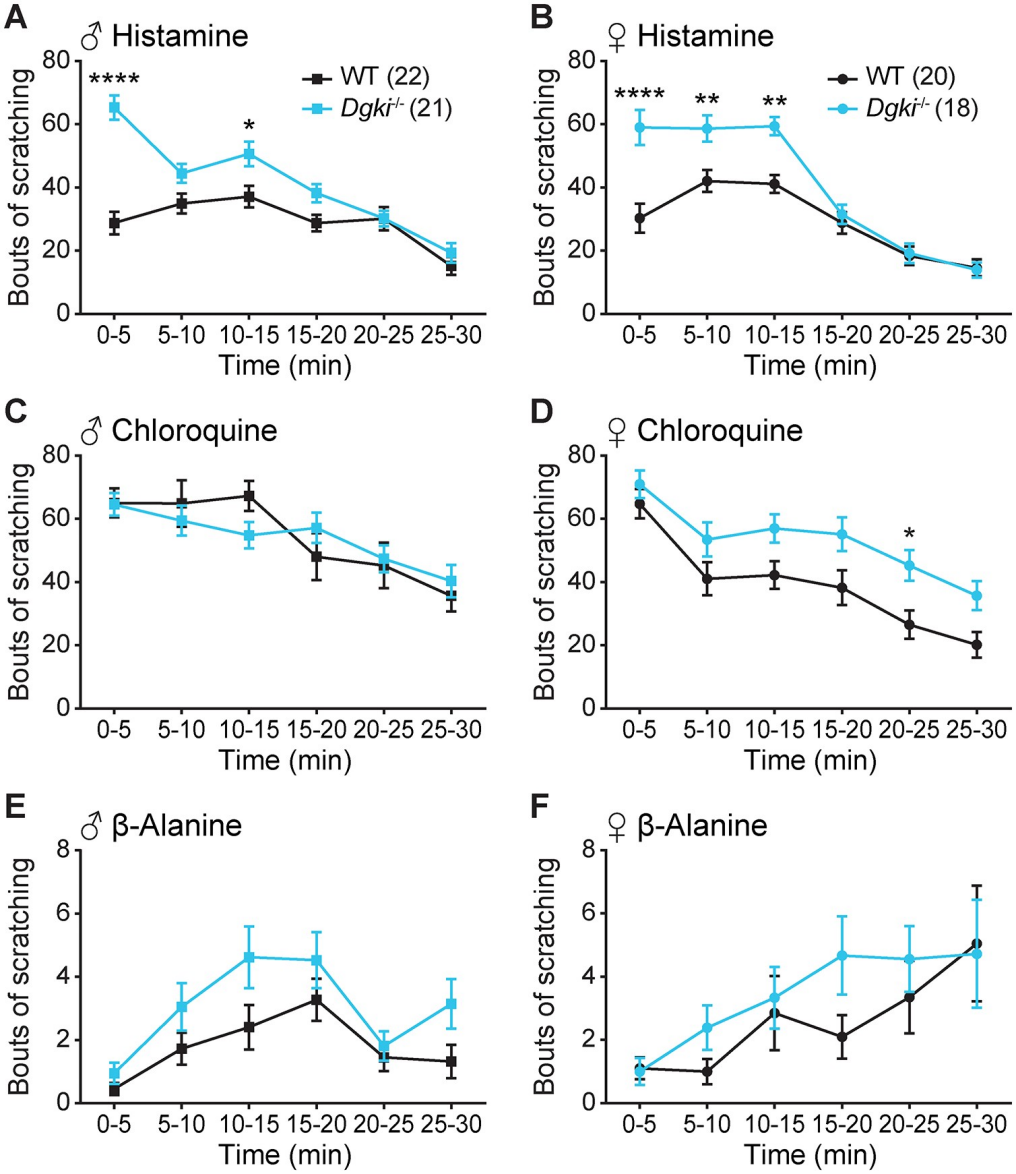
Loss of *Dgki* enhances histamine-induced scratching in male and female mice. An acute scratching assay was used to test itch responses in *Dgki*^-/-^ mice following an injection of 500 μg histamine (A-B), 200 μg chloroquine (C-D), or 500 μg β-alanine (E-F) in WT and *Dgki*^-/-^ male (A,C,E) and female (B,D,F) mice in 5 minute intervals. The number of mice of each sex tested for all pruritogens is indicated in parentheses in (A) and (B). Graphs show mean ± SEM. p< *0.05, **0.01, ***0.001, ****0.0001.

Male and female WT and *Dgki*^-/-^ mice were assayed for their scratching responses to injections of the pruritogens histamine, chloroquine, and β-alanine (Fig 2; full data in S1 Table). Histamine caused significantly greater scratching responses in both male and female *Dgki*^-/-^ mice relative to WT mice (Fig 2A and 2B). Chloroquine and β-alanine elicited scratching behavior equally in *Dgki*^-/-^ and WT animals (Fig 2C–2F). Overall, the greatest impact of *Dgki* loss was seen in both males’ and females’ responses to histamine, especially in the first 5 minutes after injection.

### Acute pain sensitivity is unaffected by *Dgki* loss in mice

**Fig 3:**
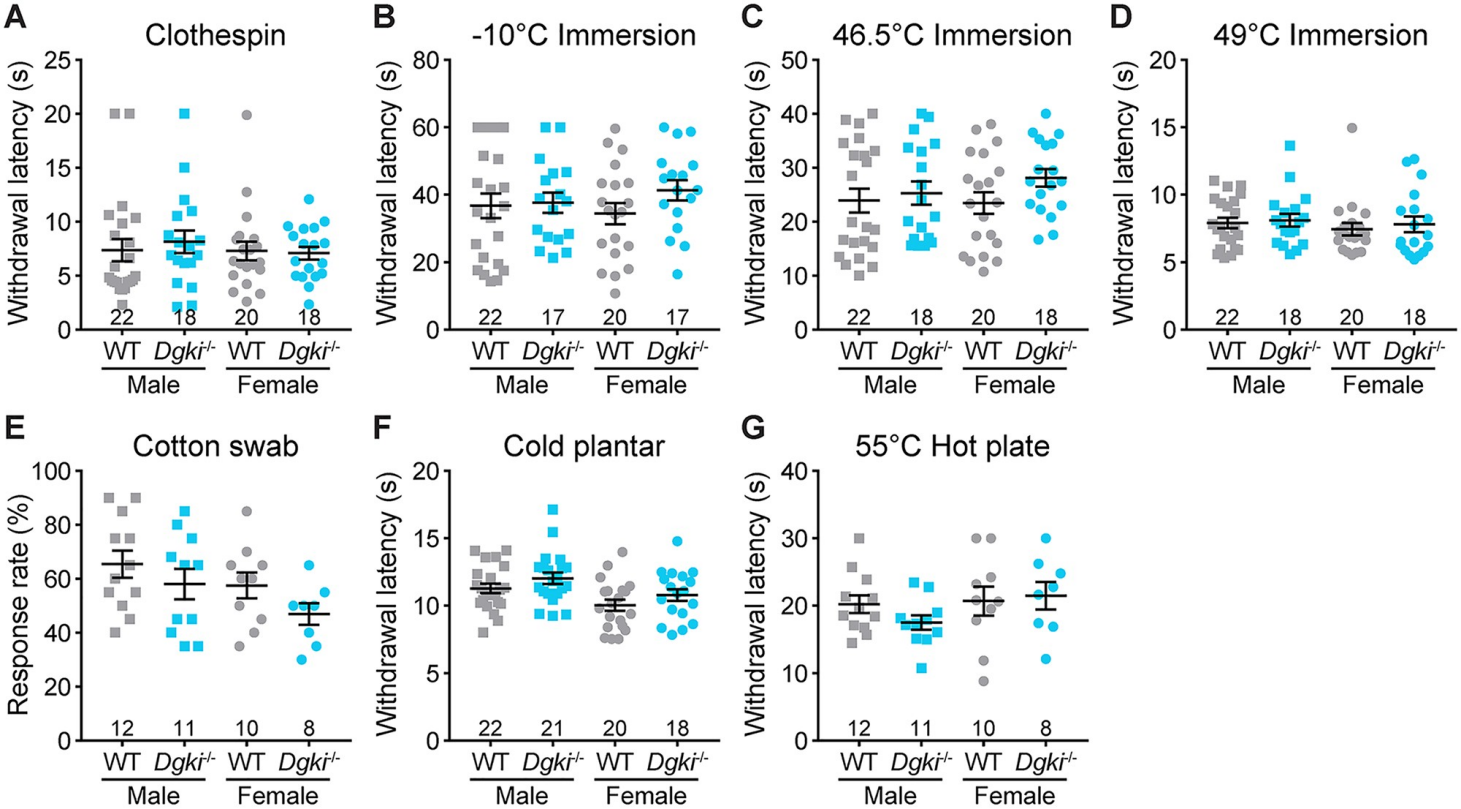
Loss of *Dgki* does not affect acute nociception in mice. (A) The latency to respond to a miniature clothespin attached to the tail. B-D) The latency to respond to immersion of the tail in a bath set to a temperature of -10°C (B), 46.5°C (C), or 49°C (D). E) The rate of response to a light touch with a fluffed-out cotton swab. F) The latency to withdraw the hindpaw from an application of dry ice. G) The latency to lick hindpaw or jump after placement on a 55°C hot plate. The number of mice tested is indicated. Graphs show mean ± SEM.

To determine if acute pain was also altered in these mice, we tested responses to thermal and mechanical stimuli applied to the tail (Fig 3A–3D) and the hindpaw (Fig 3E–3G) of WT and *Dgki*^-/-^ male and female mice. Sensitivity to noxious (Fig 3A) or innocuous (Fig 3E) mechanical stimuli was unaffected by *Dgki* deletion in either sex. Additionally, loss of *Dgki* had no effect on responses to noxious cold (Fig 3B and 3F) or noxious hot (Fig 3C, 3D and 3G) stimuli.

### Sensitization and recovery following inflammation or nerve injury are unchanged in *Dgki*^*-/-*^ *mice*

**Fig 4:**
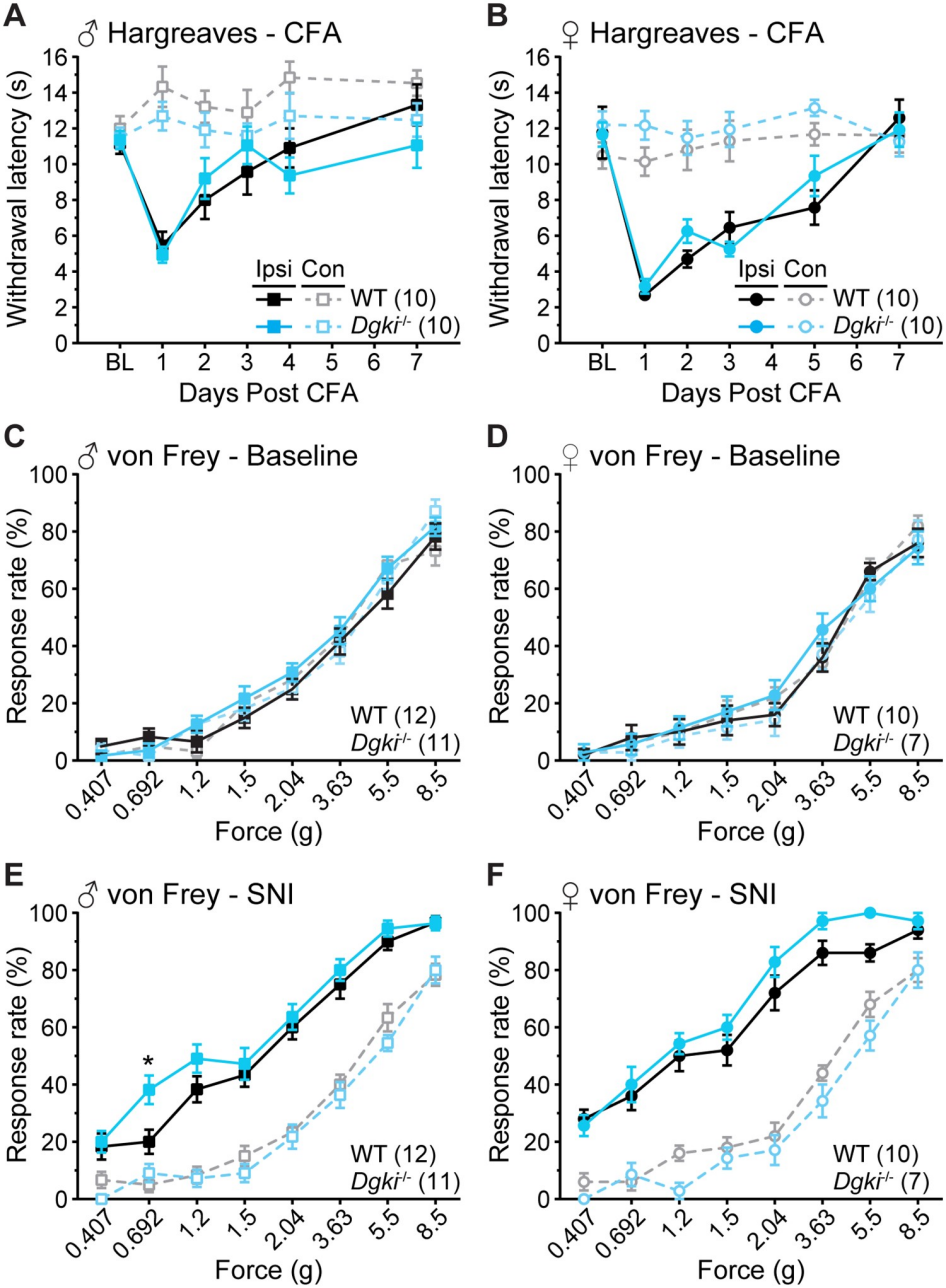
*Dgki* deletion does not alter sensitization or recovery in chronic inflammatory or neuropathic pain models. Withdrawal latencies to heat in the Hargreaves assay for 7 days following injection of complete Freund’s adjuvant (CFA) into the plantar surface of the ipsilateral and contralateral hindpaws in male (A) and female (B) *Dgki*^-/-^ and WT mice. BL = baseline. Response rates to von Frey filaments of the indicated forces in male (C,E) and female (D,F) *Dgki*^-/-^ and WT mice at baseline (C-D) and 14 days after spared nerve injury (SNI) surgery (E-F) in the ipsilateral and contralateral hindpaws. The number of mice tested is indicated in parentheses. Graphs show mean ± SEM. *p<0.05.

Although baseline pain sensitivity was not disturbed in these animals, we hypothesized that *Dgki* loss may alter sensitization to nerve or tissue injury. We analyzed responses to the CFA model of inflammatory injury, using the Hargreaves assay to demonstrate changes in thermal sensitivity (Fig 4A and 4B). In both males and females, genotype did not influence heat hypersensitivity in the ipsilateral paws. For mice of both sexes, the responses of the contralateral paw did not vary significantly over time and did not differ based on genotype.

We analyzed responses to the SNI model of neuropathic injury, using the von Frey assay to demonstrate changes in mechanical sensitivity (Fig 4C–4F). Here, we show responses of the ipsilateral and contralateral paws at baseline (Fig 4C and 4D) and on Day 14 post-surgery (Fig 4E and 4F), which is when neuropathic sensitization is well-established. Apart from a small increase in the response rate of the ipsilateral paw to a force of 0.692 g in *Dgki*^-/-^ males on Day 14, the overall degree of sensitization did not differ between WT and *Dgki*^-/-^ mice of either sex. There were no differences between ipsilateral and contralateral paws at baseline, and no changes in responses from the contralateral paw over time.

### *Dgki* loss enhances *in vitro* DRG neuronal responses to histamine but not algogens

**Fig 5:**
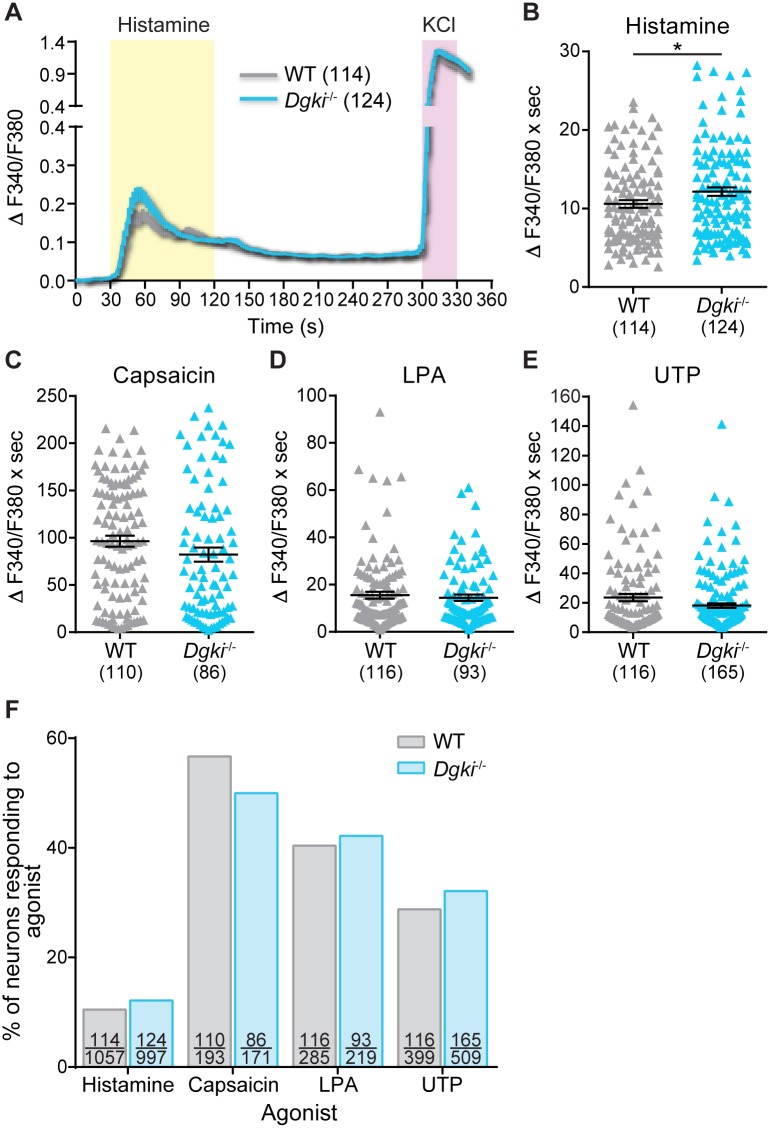
*In vitro* calcium responses to histamine, but not algogenic agonists, are enhanced in *Dgki*^*-/-*^ mouse DRG neurons. Calcium activity of DRG neurons dissected and cultured from WT and *Dgki*^-/-^ mice in response to 100 μM histamine (A,B), 1 μM capsaicin (C), 10 μM lysophosphatidic acid (LPA; D), or 100 μM uridine triphosphate (UTP; E). Each experiment ended with exposure to 100 mM KCl to ensure neuron health. Average calcium responses over time in WT and *Dgki*^-/-^ DRG neurons are shown for histamine experiments in (A). The area under the curve representing the 90 s of agonist exposure was calculated for each responding neuron, plotted in (B-E). F) The proportion of all healthy neurons (neurons respond to KCl) responding to the indicated agonists. For (A-E) the number of responding neurons is indicated in parentheses, and graphs show mean ± SEM. For (F) on each bar the top number indicates the number of neurons responding to the agonist and the bottom number indicates the total number of healthy neurons.

Because *Dgki* is highly expressed in DRG neurons, we hypothesized that altered signaling activity in these neurons caused the enhanced responses to histamine *in vivo*. Using cultured WT and *Dgki*^-/-^ DRG neurons loaded with a fluorescent ratiometric calcium dye, we measured changes in intracellular calcium concentrations induced by stimulation of receptors involved in itch and pain signaling (Fig 5). *Dgki* loss caused a small but significant increase in calcium responses to histamine in cultured DRG neurons (Fig 5A and 5B). *Dgki*^-/-^ neurons did not respond differently from WT after exposure to the algogenic compounds capsaicin, LPA, or UTP (Fig 5C–5E). The response rate—the proportion of healthy neurons responding to the agonist—did not differ between WT and *Dgki*^-/-^ cultures for all four compounds tested (Fig 5F).

## Discussion

Here, we sought to determine how *Dgki* loss in mice affects somatosensory behaviors. *Dgki* deletion significantly enhanced scratching responses to histamine, but not to other pruritogens. The effect of *Dgki* loss on somatosensory behavior was specific to histamine-induced itch, as there were no alterations in baseline pain or pain sensitization in these animals. Further, our *in vitro* calcium imaging experiments showed enhanced response magnitudes in *Dgki*^-/-^ neurons following histamine exposure. Thus, our data suggest that enhanced responses to histamine *in vivo* following *Dgki* deletion is mediated by DRG neurons, though other cells may contribute to this phenotype.

The only tissue in which *Dgki* is expressed more highly than in the DRG is in mast cells (Table 1) [41]. In response to allergen exposure mast cells release histamine, which binds receptors on nearby sensory neurons. Mast cells can respond to histamine themselves as well [47, 48]. Other *Dgk* isoforms have been shown to affect mast cell function. *Dgkz* loss decreases FcεRI-induced degranulation in bone marrow-derived mast cells but increases cytokine production [49]. Additionally, knockdown of *Dgkg*, but not *Dgka*, reduces degranulation in RBL-2H3 cells, a histamine-releasing, mast-cell-like line [50]. Future studies will be required to interrogate the function of *Dgki* in mast cells and to determine the mechanism by which *Dgki* may function to downregulate responses to histamine. Overall, our research reveals the potential of *Dgki* to mediate histamine-dependent itch, and could help to identify future therapies for patients with chronic allergen-mediated itch.

## Supporting information

S1 Table Raw data for itch assay. Raw values for number of scratches in each 5-minute window over a 30-minute period, for the data presented in Fig 2.
(XLSX)
